# Trimethoprim sulfamethoxazole drug resistance with co resistance to extended spectrum β-lactam antibiotics among bacterial isolates from HIV patients

**DOI:** 10.1186/1471-2334-14-S3-P57

**Published:** 2014-05-27

**Authors:** Marimuthu Ragavan Rameshkumar, Nallusamy Vijaykanth, Ramachandran Vignesh, Pachamuthu Balakrishnan, Suniti Solomon, Narasingam Arunagirinathan

**Affiliations:** 1 PG & Research Department of Microbiology and Biotechnology, Presidency College (Autonomous), Chennai, India; 2 YRG Centre for AIDS Research and Education, Chennai, India

## Background

Trimethoprim-sulfamethoxazole (TMP-SMX) is a broad spectrum antimicrobial agent and also reduces the mortality among adults and children when used as prophylaxis against opportunistic infections in HIV infected patients. Drug resistant to TMP-SMX along with Extended spectrum β-lactamase (ESBL) production among Enterobacteriaceae is creating major therapeutic problem in clinical settings for treating the bacterial infections among HIV individuals.

## Methods

TMP-SMX drug resistance among the isolates was identified using Kirby-Bauer disc diffusion method and ESBL production by combination disc method (CDM). Cefotaxime (30µg) and cefotaxime/clavulanic acid (30μg/10μg) discs were placed 20 mm apart on the agar surface. Similarly, the ceftazidime (30µg) and ceftazidime/clavulanic acid (30μg/10μg) discs were also placed. After incubating overnight at 37°C, a ≥ 5mm increase in the zone diameter was interpreted as positive for ESBL production. Statistical analysis was done using SPSS software version 15.0.

## Results

A total of 103(40 *Escherichia coli*, 15 *Klebsiella pneumoniae*, 13 *Pseudomonas aeruginosa*, 10 *Klebsiella oxytoca*, 8 *Proteus mirabilis*, 2 *Proteus vulgaris*, 11 *Staphylococcus aureus*, 3 *Staphylococcus epidermidis* and 1 *Streptococcus* sp.) bacterial strains were isolated from HIV patients. Among these 65(63.10%;*p*=0.008) isolates were resistance to TMP-SMX and only 40(38.83%;*p*=0.023) isolates were resistant to extended spectrum β-lactam antibiotics. Twenty nine ESBL producers from HIV patients were found to be co resistant to TMP-SMX. All ESBL producing isolates showed resistance to ceftazidime and also for ceftazidime/clavulanic acid combination.

## Conclusion

A rapid increase in the use of prophylactic TMP-SMX might be responsible for the TMP-SMX drug resistance among opportunistic bacterial infections in HIV patients.

